# What is hemoglobin, albumin, lymphocyte, platelet (HALP) score? A comprehensive literature review of HALP’s prognostic ability in different cancer types

**DOI:** 10.18632/oncotarget.28367

**Published:** 2023-02-25

**Authors:** Christian Mark Farag, Ryan Antar, Sinan Akosman, Matthew Ng, Michael J. Whalen

**Affiliations:** ^1^Department of Urology, George Washington University School of Medicine, Washington, DC 20052, USA; ^2^Department of Surgery, George Washington University School of Medicine, Washington, DC 20052, USA

**Keywords:** HALP score, biomarker, prognosis, cancer, survival

## Abstract

Since its inception, the Hemoglobin, Albumin, Lymphocyte, Platelet Score (HALP) has gained attention as a new prognostic biomarker to predict several clinical outcomes in a multitude of cancers. In our review, we searched PubMed for articles between the first paper on HALP in 2015 through September 2022, yielding 32 studies in total that evaluated HALP's association with various cancers, including Gastric, Colorectal, Bladder, Prostate, Kidney, Esophageal, Pharyngeal, Lung, Breast, and Cervical cancers, among others. This review highlights the collective association HALP has with demographic factors such as age and sex in addition to TNM staging, grade, and tumor size. Furthermore, this review summarizes HALP's prognostic ability to predict overall survival, progression-free survival, recurrence-free survival, among other outcomes. In some studies, HALP has also been able to predict response to immunotherapy and chemotherapy.

This review article also aims to serve as a comprehensive and encyclopedic report on the literature that has evaluated HALP as a biomarker in various cancers, highlighting the heterogeneity surrounding HALP's utilization. Because HALP requires only a complete blood count and albumin - already routinely collected for cancer patients - HALP shows potential as a cost-effective biomarker to aid clinicians in improving outcomes for immuno-nutritionally deficient patients.

## INTRODUCTION

In the last several years, the Hemoglobin, Albumin, Lymphocyte, Platelet Score (HALP) has emerged in the literature as a new prognostic biomarker that has been used to predict a number of clinical outcomes in the context of various neoplasms. HALP is a novel immune-nutritional marker that integrates several routinely collected indicators of immune status, such as the platelet and lymphocyte count, nutritional status, such as albumin, and hemoglobin, a marker for anemia.

Immunonutritional status is an important consideration for patients with cancer as cancer patients have increased metabolic demands and are at risk for a chronic catabolic state/cachexia, in addition to caloric deficits from the anorexia induced by systemic oncologic treatments (i.e. chemotherapy), in addition to myelosuppression and immunocompromise. To date, HALP has been previously implemented in a number of research articles evaluating outcomes in Gastric, Colorectal, Bladder, Prostate, Kidney, Esophageal, Pharyngeal, Lung, Breast, and Cervical cancers, among others. Despite the growing interest that HALP has garnered in scientific literature, to our best knowledge HALP remains largely a theoretical framework with no evidence, to date, of clinical adoption. To date, one meta-analysis has shown that low pre-treatment HALP predicts worse overall outcomes in cancer patients [1]. However, the studies investigating HALP are extremely heterogenous in cancer type, outcome, HALP threshold, and population of interest. This review article aims to summarize and report the literature surrounding HALP and its viability as a prognostic marker in various cancers in an encyclopedic manner. Studies included are from the inception of HALP in 2015 through September 2022.

### What is HALP?

HALP is an immunonutritional biomarker that has been used to predict prognosis in patients with malignancy. The HALP score is calculated as HALP Score = [hemoglobin (g/L) × albumin (g/L) × lymphocytes (/L)]/platelets (/L), [2] and was developed by Chen et al., to predict prognosis in gastric carcinoma. These four markers are essential considerations for immune and nutritional status in the context of cancer. The putative mechanisms underlying the importance of each of these components are explored below.

Platelets have been shown to play a key role in the metastatic capabilities of cancer [3]. Platelets release vascular endothelial growth factor (VEGF) and promote tumor angiogenesis [4–6], among other inflammatory mediators [7]. Platelets have also been demonstrated to have a role in protecting tumor cells from immune detection [8]. Tumor-cell-induced platelet aggregation (TCIPA) has been demonstrated in several types of cancers and has been shown to correlate with higher metastatic potential [9–11]. Platelet aggregation surrounding cancer cells results from the many adhesion molecules that platelets express, including integrins (αIIbβ3), selectins (P-selectin), leucine-rich glycoproteins (P-selectin glycoprotein ligand -PSGL-1- and GPIb/V/IX), and immunoglobulin superfamily proteins (platelet-endothelial adhesion molecule -PECAM-1) [12, 13]. TCIPA results in a coating of platelets surrounding cancer cells, preventing them from being detected by natural killer (NK) cells in mouse models [14]. Accordingly, proinflammatory states may result in thrombocytosis.

Anemia is a well-documented phenomenon that occurs in patients with cancer and may occur through several mechanisms. For example, anemia of chronic disease (ACD) is a well-documented cancer phenomenon [15]. In ACD, CD3 T lymphocytes and macrophages release pro-inflammatory cytokines such as IL-6, among others [16]. IL-6 mediates Hepcidin release by the liver and inhibits iron absorption and release of iron to prevent iron utilization by cancer cells [17, 18]. Furthermore, IL-6 is known to reduce erythropoiesis by other non-iron mediated mechanisms [15, 19]. Anemia can also occur through other cancer-specific mechanisms. For example, in colorectal and other GI tumors, the tumor may erode into the normal tissue and cause chronic bleeds resulting in iron deficiency anemia [20]. For example, gross hematuria may contribute to iron deficiency anemia in advanced urothelial carcinoma [21]. In patients who undergo radical cystectomy, the terminal ileum is used as a urinary conduit and may decrease the absorption of Vitamin B12, theoretically resulting in megaloblastic anemia (“pernicious anemia”) years after the surgery [22]. Malnutrition may also lead to megaloblastic anemia via deficiencies of Vitamins B12 and B9 and iron [23].

Albumin levels are affected by a patient’s nutritional state and metabolic demands. Inflammation and high nutritional risk have both been associated with low albumin [24]. During an inflammatory process, C-reactive protein (CRP) tends to rise, while albumin levels are noted to drop; the Glasgow Prognostic Score - a combination of CRP and Albumin - has been used in more than 60 studies to predict various outcomes in cancer including survival and chemotherapy effectiveness and tolerability [25–27]. As such, albumin levels are a well-established indicator for prognosis in various cancers [28–31]. The cancer-related anemia and hypoalbuminemia in the context of malnutrition commonly accompany cancer cachexia, which is defined as a complex multifactorial syndrome with ongoing skeletal muscle loss [32]. Increased proinflammatory cytokines, in addition to lower serum proteins like albumin, seen in cancer patients are instrumental in developing cachexia [33]. As such, high serum albumin levels have been associated with improved 1-year mortality rates in cancer patients with cachexia [34].

Lymphocytes play a role in immunosurveillance, aiding tumor detection and destruction; therefore, the depletion of lymphocyte count is thought to play an important role in prognosis [35]. Previously, other prognostic indicators such as the platelet-to-lymphocyte ratio (PLR) and neutrophil-to-lymphocyte (NLR) ratio have incorporated lymphocyte scores to aid in the prognosis of various cancers with marked success [36–40]. Furthermore, lymphocyte count has been shown to decrease while receiving cytotoxic chemotherapy via well-described myelosuppression [41].

The HALP score incorporates each of these markers of nutrition and inflammation to comment on the overall prognosis of patients with malignancies. As markers of superior immune-nutritional status, lymphocytes, hemoglobin, and albumin are factored into the numerator of the HALP score, and as an indicator of disease, platelets are factored into the denominator of the HALP calculation. As such, a high HALP score is conceptualized as a positive indicator of prognosis to detect patients who have low immune-nutritional function and provide more nuanced risk stratification.

The following sections detail the utilization of HALP for various cancers. Studies have been published from institutions around the world utilizing cohorts of various sizes. Admittedly, the primary endpoints are heterogeneous and include the prediction of lymph node metastasis, progression-free survival, and overall survival, among others. As such, there is no standardized or universal threshold of HALP score to risk stratify for these outcomes. Based on the extant literature, the meaningful HALP cutoff is disease-specific and largely study-specific.

### HALP and age

Interestingly, studies reported conflicting results regarding HALP and age. A total of eight studies showed significant differences between HALP and age. Specifically, five papers suggested that as one ages, the HALP score decreases [42–47]. In contrast, two papers found the opposite result [48, 49]. Fifteen papers reported no significant difference in age; meanwhile, eight others did not assess this relationship. Shen et al., actually found that there were no significant differences in age of the HALP score. Still, they found that HALP was only significant in older patients ≥65 years old and lost significance when considering patients of all ages [50]. It is worth noting that nearly every single paper considered the age of patients when running multivariable regressions or constructing a nomogram. It is worth re-emphasizing that cancer is often a disease of older age in solid tumor cancers.

Intuitively, as one might expect, the HALP score intrinsically decreases with age, as the hemoglobin and albumin scores decrease with age [51–53]. Furthermore, CD4+ and CD8+ T cell counts and B cell counts tend to decrease at a consistent rate as one ages [54, 55]. However, platelet counts have been shown to decrease after the age of 60 in the healthy population. In one prospective study of over 21,000 patients, a lower platelet count predicted an increased risk of developing cancer in the future [56, 57]. However, a low platelet count would increase the HALP score. Still, the fact remains that patients tend to have elevated platelet counts at the time of new cancer diagnosis, evidenced by a 10-year analysis of nearly 9 million Ontario residents, which served as an excellent representation of the general population [58]. The paradox displayed in these two large-scale population studies highlights the malleability of platelet score, and thus the HALP score, whereby a low platelet count increases the risk of developing cancer, but platelet counts then rise rapidly by the time of diagnosis. Ultimately, we are limited by the fact that no one has studied the HALP score in a subset of healthy patients to properly assess age-related changes in the HALP score.

### HALP and sex

One important consideration with HALP is the role that sex may play in calculating HALP scores due to differences in the reference values for males and females. The accepted minimum hemoglobin scores in females are lower at baseline than in males, which inherently means that males will have higher HALP scores at baseline [59].

The first paper to consider HALP by Chen et al., attempted to address this with a correction, adding 10 g/L to the hemoglobin score of every female. Still, the X-tile calculated optimum cutoff did not change significantly (56.6 vs. 56.8) [2]. As such, the study proceeded to directly use the value of hemoglobin in calculating HALP, irrespective of sex. In total, five different studies showed statistically significantly lower HALP scores in females than in males [2, 42, 43, 47, 60]. These papers simply adjusted for sex in their multivariable models. It is worth noting that several papers only looked at patients of single sex. For example, papers on breast, endometrial, and cervical cancer only considered female patients, while papers on prostate cancer and one paper on esophageal cancer exclusively male populations [48, 61–66]. Two papers on pharyngeal cancer had primarily male populations [49, 67]. Amongst those with female only populations, three of the four papers showed HALP to have prognostic benefit, with only Njoku et al., not concluding in favor of HALP. To account for the potential difference in HALP, Feng et al., ran two separate Kaplan-Meier analyses for males and females, and found both to be statistically significant when predicting cancer-specific survival, despite using the same HALP cutoff for both subsets.

Several studies reported differences in baseline HALP between males and females, but even when adjusting for sex, HALP maintained significance. In other words, while a difference in HALP score may exist between males and females, the current results suggest that it does not significantly impact HALP’s utility as a biomarker.

### Gastric cancer (Table 1)

The seminal research article to develop HALP as a prognostic tool was published in 2015 by Chen et al., in which the authors investigated the survival of gastric carcinoma patients following gastrectomy [2]. This paper utilized X-tile software (Yale Lab) to determine an optimal HALP score threshold score of 56.8. Those with a HALP score above 56.8 were shown to have statistically significantly improved overall survival compared to those who scored less than 56.8 using both Kaplan Meier and Cox Regression, both in the training set (*n* = 888) and validation set (*n* = 444) of patients. Patients with High HALP were also shown to have a smaller tumor size and to be more likely to stage T1-T2, N0, and TNM 1A. However, even when these features were controlled for in multivariable Cox Regression, HALP remained a significant predictor of overall survival. In addition to predicting tumor stage, this paper was also the first to demonstrate that HALP could potentially differentiate the prognosis of patients within the same tumor stage. However, this paper could only show this in the training set, in which those with TNM Stage III had improved overall survival in Kaplan-Meier analysis (*p* = 0.030) but lost significance in the validation set (*p* = 0.231). No other TNM Staging Group stratification was significant. When considering HALP as a continuous variable, HALP was significant in both univariable and multivariable Cox Regression. Nomograms based on HALP score were more accurate in prognostic prediction than TNM staging alone.

**Table 1:** Gastric and other gastrointestinal cancers (*N* = 4)

Study name; author (year)	Study type/size	HALP threshold (method)	Outcome(s)	Summary
Prognostic significance of the combination of preoperative hemoglobin, albumin, lymphocyte and platelet in patients with gastric carcinoma: a retrospective cohort study; Chen et al., (2015)	Retrospective *N* = 1332	56.8 (X-tile)	OS in Gastric Cancer patients who underwent gastrectomy.	Patients who had high HALP ≥56.8 were shown to have smaller tumor size and less advanced tumor stage. High HALP patients also had superior overall survival. HALP was more accurate in prognostic prediction than TNM stage alone.
A novel robust nomogram based on preoperative hemoglobin and albumin levels and lymphocyte and platelet counts (HALP) for predicting lymph node metastasis of gastric cancer; Wang et al., (2021)	Retrospective *N* = 349	35.3 (ROC)	Predicting Lymph node metastasis of gastric cancer.	HALP was an independent risk factor for lymph node metastasis, which was used as part of a nomogram in addition to alcohol use, depth of invasion, differentiation, CEA, and CA19-9.
Haemoglobin, albumin, lymphocyte and platelet predicts postoperative survival in pancreatic cancer; Xu et al., (2020)	Prospective *N* = 582	44.56 (X-tile)	OS and RFS in pancreatic adenocarcinoma who underwent radical resection.	Patients with HALP <44.56 were found to be more likely to have lymph node metastasis, poor tumor differentiation, and high TNM staging. Low HALP was also a significant predictor of worse overall survival and worse recurrence-free survival.
Comparison of Prognostic Value of Red Cell-Related Parameters of Biliary Tract Cancer After Surgical Resection and Integration of a Prognostic Nomogram: A Retrospective Study; Sun et al., (2021)	Retrospective *N* = 418	42.68 (X-tile)	OS in surgical resection in patients with biliary tract cancer – integrated into a prognostic nomogram.	HALP > 42.68 an independent predictor of better overall survival, and outperformed other parameters tested. Nomogram which included both TNM Staging and HALP outperformed TNM alone.

In 2021, Wang et al., reported on the prognostic ability of HALP in gastric cancer, utilizing HALP in a nomogram to predict lymph node metastasis in gastric cancer [68]. Using a training set of 250 patients, an optimum cutoff for HALP of 35.3 was determined utilizing receiver operating characteristics (ROC), in which the area under the curve (AUC) for HALP was 0.644 (sensitivity 69.1% and specificity 65.3%). On ROC, HALP individually outperformed Prothrombin Time, carcinoembryonic antigen (CEA), and carbohydrate antigen 19-9 (CA199). Furthermore, patients with lymph node metastasis were shown to have significantly lower HALP scores (27.2 vs. 38.8, *p* < 0.001). Patients with HALP ≤ 35.3 were over four times more likely to have lymph node metastasis on univariable analysis and over two times more likely on multivariate analysis. The final nomogram, which included alcohol use, CEA, tumor differentiation, HALP, CA199, and depth of tumor invasion, displayed an AUC of 0.854.

Among these studies investigating gastric cancers, the HALP score cutoff range was between 35.3–56.8, with a median of 46.05. The median HALP score across all cancer subtypes in our review was 31.2. This shows that the gastric cancer HALP cutoff is higher than the median of all cancer subtypes.

### Other gastrointestinal cancers (Table 1)

Beyond Gastric and Colorectal Cancer, HALP has also been investigated in other gastrointestinal cancers. In 2020, Xu et al., investigated the prognostic ability of HALP on 582 radically resected pancreatic adenocarcinoma patients [60]. X-tile software was used to find an optimal cutoff of 44.56. Patients with HALP <44.56 were found to be more likely to have lymph node metastasis (*p* = 0.002), poor tumor differentiation (*p* = 0.032), and high TNM staging (*p* = 0.008). Low HALP was also a significant predictor of overall survival and recurrence-free survival on both univariable and multivariable Cox regression analysis. Patients with high HALP had a median OS of 23.6 months versus 11.5 months, and patients with high HALP had a median RFS of 16.3 months vs. 7.3 months. Patients with pancreatic head tumors were found to have lower HALP than patients with pancreatic body/tail tumors (p<0.001). Still, HALP was significant in predicting both RFS and OS, irrespective of whether the tumor was located in the pancreatic head or body/tail. It is noteworthy that this is the only study to date on HALP and pancreatic cancer.

A 2021 Sun et al., analyzed 418 biliary tract cancer patients and showed that low HALP was associated with worse overall survival, outperforming other hematological markers [69]. X-tile was used to find an optimal cutoff of 42.68. Multivariable Cox analysis showed that low HALP, TNM staging, and non-radical resection were all independent factors associated with worse overall survival. Of note, the authors also used these variables to construct a predictive nomogram for overall survival, which was shown to outperform the traditional AJCC TNM System alone.

Both gastrointestinal cancer studies reported a HALP cutoff range of 42.68-44.56 with a median score of 43.62, which is higher than the median HALP cutoff of 31.2 in our review.

### Colorectal cancer (Table 2)

HALP has also been studied in colorectal cancers, initially in 2016 by Jiang et al., in which the authors prospectively sought to predict survival in patients with locally advanced colorectal cancer (LACRC) who underwent radical resection [70]. In this paper, X-tile software determined an optimal threshold of 26.5. Patients who had a HALP >26.5 were found to have statistically significantly better 5-year overall survival (OS) and cancer-specific survival (CSS) in Cox regression multivariable analysis, adjusting for gender, tumor location, smoking, alcohol history, age, family history, differentiation grade, vessel/nerve invasion, and TNM staging. Furthermore, in both the training set (*n* = 684) and validation set (*n* = 136), Kaplan Meier analysis also significantly improved OS and CSS in those with HALP >26.5.

**Table 2:** Colorectal summary (*N* = 5)

Study name; author (year)	Study type/size	HALP threshold (method)	Outcome(s)	Summary
Preoperative combined hemoglobin, albumin, lymphocyte and platelet levels predict survival in patients with locally advanced colorectal cancer; Jiang et al., (2016)	Prospective *N* = 820	26.5 (X-tile)	OS and CSS in locally advanced colorectal cancer.	Low HALP conferred worse prognostic 5-year OS and CSS in patients with locally advanced colorectal cancer.
Prognostic significance of preoperative hemoglobin and albumin levels and lymphocyte and platelet counts (HALP) in patients undergoing curative resection for colorectal cancer; Yalav et al., (2021)	Retrospective *N* = 279	15.7 (ROC)	OS and Post-operative complications.	HALP score > 15.7 is closely related to clinic pathological features and is an independent prognostic factor for overall survival. Its value in estimating mean survival is limited. Postoperative outcomes were similar between groups.
The Efficacy of Hemoglobin, Albumin, Lymphocytes, and Platelets as a Prognostic Marker for Survival in Octogenarians and Nonagenarians Undergoing Colorectal Cancer Surgery; Dagmura et al., (2021)	Prospective *N* = 139	15.5 (ROC)	OS in patients undergoing curative colorectal cancer surgery.	Low HALP biomarker was associated with worse prognosis of patients treated surgically for colorectal cancer with curative intent. Furthermore, HALP score was significantly different in octogenarians compared to their younger counterparts.
Can HALP (Hemoglobin, Albumin, Lymphocytes, and Platelets) Score Differentiate Between Malignant and Benign Causes of Acute Mechanic Intestinal Obstruction?; Akbas et al., (2021)	Retrospective *N* = 192	23.94 (ROC)	Benign vs. Malignant causes of acute mechanical intestinal obstruction.	HALP <23.94 was an independent prognostic variable for predicting malignant causes of acute mechanical intestinal obstruction.
Diagnostic Value of Preoperative Haemoglobin, Albumin, Lymphocyte and Platelet (HALP) Score in Predicting Tumour Budding in Colorectal Cancer; Topal et al., (2022)	Retrospective *N* = 110	31.6 (ROC)	Predicting the presence or absence of tumor budding in patients undergoing surgery.	HALP score was not statistically significantly associated with the presence or absence of tumor budding.

In 2021, Yalav et al., used HALP to predict survival following curative surgery for colorectal cancer in 279 patients retrospectively [71]. However, this study used ROC and found a lower threshold of 15.73. In this study, patients with low HALP <15.73 were found to have higher CEA levels, more likely to be mucinous adenocarcinoma, and more likely to be poorly differentiated. Furthermore, HALP was a statistically significant predictor of overall survival. This study also sought to investigate if HALP could predict postoperative complications according to the Clavien-Dindo classification. However, this investigation was negative. This study also concluded that while HALP is an independent prognostic factor for survival, its value in estimating mean survival is limited, with a sensitivity of 45.4% and a specificity of 66.9%.

Another study by Dagmura et al., in 2021 prospectively investigated survival in colorectal surgery patients and found similar results, using ROC to find an optimum threshold of 15.5 (AUC = 0.775, sensitivity 78.2%, specificity 69%) [46]. Patients with a HALP ≥15.5 had a statistically significantly longer overall survival than patients <15.5 (2685 days vs. 1306 days, *p* < 0.001). Furthermore, HALP as a continuous variable was a significant predictor on both univariate and multivariate Cox regression analysis adjusting for age. Interestingly, this study also determined that HALP scores were higher for older patients compared to younger patients (19 vs. 14, *p* = 0.047). HALPs predictive value was improved by stratifying patients by age.

Akbas et al., used HALP to differentiate between malignant and benign causes of acute mechanical intestinal obstruction [72]. In this study, 192 patients underwent surgical treatment for mechanical obstruction. In 80 benign obstruction patients, the mean HALP score was 39.33, but in 112 patients with malignant causes of obstruction, the mean HALP score was 16.98 (*p* < 0.001). ROC was also used to determine an optimal threshold of 23.94 (AUC = 0.86, sensitivity 85%, specificity 78%). HALP also showed predictive ability of malignant causes of mechanical intestinal obstruction on binary logistic regression (*p* < 0.001). However, this study did not consider the prognostic ability of HALP.

Most recently in 2022, Topal et al., assessed HALP as a predictor of tumor budding in colorectal cancer [73]. Tumor budding is considered a histological reflection of epithelial-mesenchymal transition and is associated with lymph node metastasis, local recurrence, and distant metastatic disease. This study examined the tumor-containing histological slides for 110 patients but found that the HALP score was not statistically significantly associated with the presence of tumor budding (*p* = 0.494). The ROC curve suggested a cutoff score of 31.6 with an AUC of 0.546 (sensitivity 70.89%, specificity 48.39%). This study did not consider the prognostic ability of HALP.

In colorectal cancer subtypes, the range of HALP score cutoffs was between 15.5–31.6 with a median of 23.94, which is lower than the median score cutoff of 31.2 in our review.

### Lung cancer (Table 3)

The first paper evaluating the prognostic ability of HALP in lung cancer was published by Shen et al., in 2019 [50]. The authors showed that in a sample of 178 retrospective small cell lung cancer patients treated with etoposide, patients with Low HALP had significantly worse progression-free survival in the short term of 6 months. X-tile was used to determine an optimal cutoff of 25.8. In Kaplan Meier analysis, the PFS of the High HALP group was significantly longer than the Low HALP group (*p* = 0.0036). The median PFS for low HALP was 5.3 months, while the median PFS for High HALP was 7.0 months (*p* = 0.004). On univariable Cox regression analysis, HALP was significant. However, on multivariable analysis, HALP lost significance when considering patients of all ages. However, HALP was an independent predictor on multivariable analysis in a subpopulation of patients ≥65 years old, in which low HALP patients were over two times more likely to have disease progression within six months (*p* = 0.014).

**Table 3:** Lung summary table (*N* = 4)

Study name (year)	Study type/size	HALP threshold (method)	Outcome(s)	Summary
The Hemoglobin, Albumin, Lymphocyte, and Platelet (HALP) Score in Patients with Small Cell Lung Cancer Before First-Line Treatment with Etoposide and Progression-Free Survival; Shen et al., (2019)	Retrospective *N* = 178	25.8 (X-tile)	PFS following first-line etoposide for SCLC.	In SCLC patients ≥65 years, a HALP score >25.8 was an independent predictor of improved outcome, associated with increased PFS for SCLC.
Hemoglobin, albumin, lymphocyte, and platelet score and neutrophil- to-lymphocyte ratio are novel significant prognostic factors for patients with small-cell lung cancer undergoing chemotherapy; Yang et al., (2020)	Retrospective *N* = 335	18.6 (X-tile)	OS in SCLC patients undergoing chemotherapy.	Low HALP (≤18.6) was an independent prognostic factor of worse OS for SCLC patients undergoing chemotherapy.
Predictive value of the hemoglobin, albumin, lymphocyte, and platelet (HALP) score and lymphocyte-to- monocyte ratio (LMR) in patients with non-small cell lung cancer after radical lung cancer surgery; Zhai et al., (2021)	Prospective *N* = 238	48.0 (ROC)	OS in NSCLC after radical lung cancer surgery.	Patients with High HALP > 48.0 demonstrated superior OS to Low HALP patients in NSCLC following radical lung cancer surgery.
The preoperative hemoglobin, albumin, lymphocyte, and platelet score is a prognostic factor for non-small cell lung cancer patients undergoing adjuvant chemotherapy: a retrospective study; Wei et al., (2022)	Retrospective *N* = 362	48.2 (OS) 53.3 (DFS) (X-tile)	OS and DFS in NSCLC patients undergoing adjuvant chemotherapy.	Low HALP <48.2 and <53.3 predicted poorer OS (*P* = 0.02) and DFS (*P* < 0.01) outcomes respectively. Furthermore, we performed stratification analysis by tumor node metastasis (TNM) stage, and the result indicated a low HALP score predicted poor OS (*P* = 0.01) and DFS (*P* = 0.04) outcomes in stage III–IV NSCLC patients.

In 2020, Yang et al., conducted a retrospective study of 335 patients with small-cell lung cancer undergoing chemotherapy. They found that low HALP was associated with worse overall survival in both univariable and multivariable analyses when adjusting for tumor staging, radiotherapy, and neutrophil-to-lymphocyte ratio [74]. This study used X-tile to determine an optimum HALP cutoff of 18.6.

HALP’s prognostic ability has also been evaluated in non-small cell lung cancer. In 2021, Zhai et al., published a prospective study of 238 patients who underwent radical lung cancer resection and found that patients with a high HALP score had better overall survival than those with a low HALP score (*p* < 0.001) [44]. A threshold of 48.0 was determined using ROC, with an AUC = 0.666 (*p* < 0.001). HALP was a significant predictor of overall survival on both univariable and multivariable analysis, adjusting for HALP, lymphocyte-to-monocyte ratio, lymph node metastasis, and degree of tumor differentiation. Interestingly, this paper found that if stratified by tumor type, low HALP was predictive of worse overall survival for lung adenocarcinoma on Kaplan Meier analysis (*p* < 0.001) but lost significance when stratifying for non-adenocarcinoma disease (*p* = 0.194).

Most recently in 2022, Wei et al., showed HALP to be retrospectively predictive in 362 patients with non-small cell lung cancer undergoing adjuvant chemotherapy [75]. Kaplan Meier analysis showed that HALP <48.2, calculated by X-tile, predicted worse overall survival (*p* = 0.02) and worse disease-free survival (*p* < 0.01). Notably, a high HALP score was also associated with a smaller tumor size (*p* = 0.009). HALP was a significant predictor for OS and DFS on univariable Cox regression analysis. On multivariable Cox regression, adjusting for age, gender, pathological tumor stage, tumor size, and lymph node metastasis, HALP was predictive for both OS (*p* = 0.048) and DFS (0.012). When stratified by TNM staging, HALP was not a significant predictor for OS and DFS in stage I-II NSCLC. However, in stage III-IV NSCLC, low HALP was predictive for worse overall survival in OS (*p* = 0.01) and DFS (*p* = 0.04).

Among these lung cancer studies, the range of HALP score cutoffs was between 18.6–48.2 with a median of 36.9, which is higher than the median score cutoff of 31.2 in our review.

### Bladder/urothelial cancer (Table 4)

In urologic cancers, HALP was first evaluated by Peng et al., in a retrospective study of 516 bladder cancer patients treated with radical cystectomy [42]. A HALP cutoff score of 22.2 was defined as optimal by utilizing the X-Tile software (Yale Lab). Using both Kaplan Meier and Cox Regression, study participants that had a HALP score <22.2 were associated with statistically significant decreased overall survival. The authors used a nomogram that included the multivariate variables of age, TNM, and ASA to further evaluate HALP’s predictive accuracy compared to TNM stage. A HALP-based risk model’s predictive accuracy was shown to be better than a TNM-based risk model, with C-indices as 0.76 ± 0.039 and 0.708 ± 0.041, respectively). Furthermore, lower HALP scores were associated with older age, female sex, high TNM stage, and ASA grade. Patients with lower HALP scores were also shown to have decreased overall survival.

**Table 4:** Bladder/urothelial cancer summary (*N* = 6)

Study name (year)	Study type/size	HALP threshold (method)	Outcome(s)	Summary
Prognostic significance of HALP (hemoglobin, albumin, lymphocyte and platelet) in patients with bladder cancer after radical cystectomy; Peng et al., (2018)	Retrospective *N* = 516	22.2 (X-Tile)	OS in urothelial cancer patients undergoing radical cystectomy.	Patients with HALP <22.2 were associated with statistically significant decreased overall survival.
Prognostic significance of the combination of preoperative hemoglobin and albumin levels and lymphocyte and platelet counts (HALP) in patients with renal cell carcinoma after nephrectomy; Peng et al., (2018)	Retrospective *N* = 1360	31.2 (X-Tile)	CSS in renal cell carcinoma patients undergoing nephrectomy.	HALP was an independent predictor of cancer-specific survival and had better prognostic predictive accuracy for cancer-specific survival when combined with the TNM system.
A HALP score-based prediction model for survival of patients with the upper tract urothelial carcinoma undergoing radical nephroureterectomy; Gao et al., (2022)	Retrospective *N* = 533	28.67 (Youden Index)	OS and PFS in non-metastatic upper tract urothelial carcinoma.	Pre-treatment HALP score can be an independent prognostic factor of overall survival and progression free survival in addition to differentiating pathologic T stages.
HALP Score as a New Prognostic Index in Metastatic Renal Cell Cancer; Ekinci et al., (2022)	Retrospective *N* = 123	0.277 (ROC)	OS in metastatic renal cell cancer patients.	HALP scores <0.277 had worse overall survival.
Platelet-to-Lymphocyte Ratio Predicts the Efficacy of Pembrolizumab in Patients With Urothelial Carcinoma; Kurashina et al., (2022)	Retrospective *N* = 54	30.05 (ROC)	OS in urothelial carcinoma patients taking pembrolizumab.	HALP scores <30.05 were found to have worse overall survival. However, PLR was determined to be a more significant independent predictor of overall survival than HALP.
Integrative Analysis of Peripheral Blood Indices for the Renal Sinus Invasion Prediction of T1 Renal Cell Carcinoma: An Ensemble Study Using Machine Learning-Assisted Decision-Support Models; Li et al., (2022)	Retrospective *N* = 1229	No HALP threshold defined.	Determined a risk model for stratifying patients with renal cell carcinoma and predicting renal sinus invasion.	HALP score used as a variable in a novel machine-learning risk model to predict renal sinus invasion.

In 2018, another study by Peng et al., found that HALP scores had better prognostic accuracy for cancer-specific survival when combined with the TNM system [43]. This study included 1,360 renal cell carcinoma (RCC) patients that had undergone nephrectomy between 2001 and 2010. An optimal cutoff of 31.2 was determined using the X-Tile Software (Yale Lab). Patients with HALP scores ≤31.2 were strongly correlated with being female, older age, a high Fuhrman grade, high TNM stage, presence of sarcomatoid transformation, tumor necrosis and lymphovascular space invasion. On univariate analysis, low HALP scores were statistically significantly associated with reduced cancer-specific survival. On multivariate analysis, high Fuhrman grade and advanced TNM stage were associated with poor prognosis. Using these multivariate variables along with HALP, the authors showed that a HALP-based risk model (C-index of 0.881 (95% CI: 0.853–0.909)) had better prognostic predictive accuracy than a TNM-stage-based nomogram (C-index 0.846 (0.812–0.880)). This study concluded that HALP was an independent predictor of cancer-specific survival for RCC patients undergoing nephrectomy and that HALP could be further utilized to predict clinical outcomes.

In 2022 Gao et al., investigated HALP for a prediction model for the survival of patients with non-metastatic upper tract urothelial carcinoma following radical nephroureterectomy [45]. After retrospectively enrolling 533 patients from two centers, this study showed that a pre-treatment HALP score could be an independent prognostic factor of overall survival and progression-free survival in addition to differentiating pathologic T stages. The optimal cutoff for HALP scores was 28.67 after performing a ROC curve analysis using the Youden index. Kaplan–Meier analysis and log-rank test showed that a HALP score below 28.67 was significantly associated with lower overall survival and progression-free survival. Furthermore, multivariate analysis revealed that a lower HALP score (<28.67) was an independent risk factor for OS (*p* = 0.006) and PFS (*p* = 0.020). The authors conducted a subgroup analysis to assess the prognostic value of HALP with pathologic T stages (pT1-4). pT1-2 patients with HALP scores below 28.67 were shown to have significantly lower overall survival than those with HALP scores above 28.67 (*p* = 0.03 for pT1, *p* = 0.049 for pT2). Progression-free survival in this subgroup did not significantly differ. In patients with pT3-4 stage tumors and HALP scores below 28.67, progression-free survival was significantly worse (*p* = 0.02 for pT3) compared to patients with HALP scores above 28.67. The pT3 and pT4 patients with HALP scores below 28.67 had lower overall survival trends (*p* = 0.055 and *p* = 0.060, respectively). However, these differences were not statistically significant due to the small sample size. This study also incorporated HALP into nomograms of OS and PFS, which determined HALP scores to be a more accurate predictor of prognosis than without it.

Additionally, in 2022 Ekinci et al., analyzed 123 metastatic renal cell carcinoma patients and found a HALP score cutoff of 0.277 using the ROC curve [76]. The authors found that patients with low HALP scores had worse overall survival with mean OS of low HALP scores being 17.7 months compared to high HALP scores being 89.7 months (*p* = 0.001), which was statistically significant in univariate and multivariate analysis as well.

HALP has also been utilized to evaluate the prognosis of a cancer drug. A study by Kurashina et al., aimed to predict pembrolizumab’s efficacy in 54 patients with advanced metastatic urothelial cancer previously treated with systemic chemotherapy [77]. A HALP score cutoff was set at 30.05 using the ROC curve and patients with HALP scores below this cutoff were found to have worse overall survival. However, in univariate and multivariate analyses, PLR was determined to be a more significant independent predictor of overall survival than HALP.

Li et al., even developed a novel machine-learning risk model that included HALP score as a variable to stratify 1,220 patients with T1 stage renal cell carcinoma and predict renal sinus invasion [78]. This study highlights the value of using HALP for establishing risk models for the early detection of various cancers.

The range of HALP score cutoffs in bladder and urothelial cancers was between 22.2–31.2 with one study reporting a cutoff score of 0.277. The median for these bladder/urothelial cancer studies was 28.67, which is lower than the median score cutoff of 31.2 in our review.

### Prostate cancer (Table 5)

To date, HALP has been evaluated in two separate studies in the context of prostate cancers, reaching separate findings. In a 2019 study by Guo et al., the authors investigated pre-operative HALP scores in patients with oligometastatic prostate cancer after cytoreductive radical prostatectomy [66]. This study utilized the X-tile software and determined an optimal HALP score cutoff of 32.4. In this paper, a low HALP score was significantly associated with a lower prostate-specific antigen progression-free survival in both metastatic and oligometastatic prostate cancer subgroups. It is important to note that systemic treatment was not standardized in this cohort, and 56.1% of patients received neoadjuvant androgen deprivation therapy. Also, these patients not only had metastatic disease, but the majority had locally-advanced disease as well (rate of pT3-4 86.7%, pN+ 47.6%, positive margins in 68.3%).

**Table 5:** Prostate cancer summary (*N* = 2)

Study name (year)	Study type/size	HALP threshold (method)	Outcome(s)	Summary
The Hemoglobin, Albumin, Lymphocyte, and Platelet (HALP) Score is a Novel Significant Prognostic Factor for Patients with Metastatic Prostate Cancer Undergoing Cytoreductive Radical Prostatectomy; Guo et al., (2019)	Retrospective *N* = 82	32.4 (X-Tile)	PFS in metastatic prostate cancer patients receiving cytoreductive radical prostatectomy.	HALP scores below 32.4 were significantly associated with a lower prostate-specific antigen progression-free survival in both metastatic and oligometastatic prostate cancer subgroups.
HALP score and albumin levels in men with prostate cancer and benign prostate hyperplasia; Kaya et al., (2020)	Retrospective *N* = 225	49.43 (Median in BPH patients) 51.2 (Median in PCa patients)	Predicting PCa using HALP.	HALP was not found to be a significant prognostic index for patients with PCa.

In a subsequent 2020 study, Kaya et al., evaluated 225 men with benign prostate hyperplasia (BPH) and prostate cancer (PCa) [65]. This study sought to use HALP to differentiate between Pca patients and those with BPH only, and thus as a cancer biomarker. This study found the Pca group to have higher HALP scores than the BPH group with median HALP scores of 51.2 and 49.43, respectively. However, this was not found to be statistically significant. Therefore, this report found no preoperative diagnostic significance of HALP scores in Pca patients. However, this study did not evaluate overall survival or progression-free survival.

In prostate cancer, the range of HALP score cutoffs was between 32.4–51.2 with a median of 41.8, which is higher than the median score cutoff of 31.2 in our review.

### Gynecologic cancer (Table 6)

HALP has also been used to evaluate the clinical outcomes of patients with various gynecological cancers. Leetanaporn et al., aimed to investigate HALP scores’ potential in predicting survival in 1,533 patients with locally advanced cervical cancers [48]. In this 13-year retrospective study, X-Tile (Yale Lab) was used to determine the HALP optimal cutoff of 22.2. Patients with HALP scores <22.2 were associated with higher stage and tumor size in addition to being independently associated with worse PFS and OS on univariate (Kaplan Meier and log-rank analysis) and multivariate analysis (Cox hazard regression model). It is worth noting that HALP scores <22.2 were associated with younger age (*p* < 0.001) and had a higher chance of receiving radiation therapy alone rather than concurrent chemoradiation therapy (*p* < 0.001). Furthermore, an ROC analysis of PFS and OS prediction was conducted with two Cox hazard regressions with and without HALP from multivariate analysis factors. The results found that incorporating HALP into the model improved the AUCs for prediction of PFS at five years from 0.71 to 0.72 in the training set (*p* < 0.001) and from 0.69 to 0.71 in the test set (*p* < 0.001). The AUCs for the prediction of OS also improved from 0.70 to 0.72 in the training set (*p* < 0.001) and 0.63 to 0.72 in the test set (*p* < 0.001). Additionally, the authors found that the detrimental effect of HALP scores below 22.2 on PFS and OS diminished after more than five years and three years survival, respectively. However, this finding is limited because the authors did not present the data for this test in the study.

**Table 6:** Gynecological cancer summary (*N* = 2)

Study name (year)	Study type/size	HALP threshold (method)	Outcome(s)	Summary
Predictive Value of the Hemoglobin- Albumin-Lymphocyte-Platelet (HALP) Index on the Oncological Outcomes of Locally Advanced Cervical Cancer Patients; Leetanaporn et al., (2022)	Retrospective *N* = 1588	22.2 (X-Tile)	OS and PFS in locally advanced cervical cancer patients undergoing RT and CCRT.	Patients with HALP scores <22.2 were associated with younger age, higher stage and tumor size in addition to being independently associated with worse PFS and OS.
Impact of pre-treatment prognostic nutritional index and the haemoglobin, albumin, lymphocyte and platelet (HALP) score on endometrial cancer survival: A prospective database analysis; Njoku et al., (2022)	Prospective *N* = 439	24 (Compiled based on various other studies)	OS, PFS, and CSS in endometrial cancer patients.	HALP scores were not shown to be associated with overall, cancer-specific or recurrence-free survival.

Furthermore, HALP scores have been used in endometrial cancer patients. In a prospective study by Njoku et al., on 439 endometrial cancer patients, HALP was specifically associated with FIGO staging, histology, disease grade, LVSI, and deep myometrial invasion [63]. However, HALP scores were not shown to be associated with overall, cancer-specific or recurrence-free survival, which this study concluded could be due to unaccounted confounders affecting HALP.

The range of HALP score cutoffs in gynecologic cancers was between 22.2–24 with a median of 23.1, which is lower than the median score cutoff of 31.2 in our review.

### Breast cancer (Table 7)

In 2022, Duran et al., investigated 307 surgical breast cancer patients to see if HALP could predict the presence of Axial Lymph Node Involvement [61]. A cutoff of 29.01 was found using an ROC curve, which predicted ALN involvement with 84.3% sensitivity, but only 26.1% specificity. The authors concluded that HALP alone is unsuitable for predicting ALN involvement, although Low HALP was associated with more advanced tumor and axillary lymph node positivity. Patients with low HALP had ALN involvement 67.7% vs. 53.3% for high HALP (*p* = 0.038). This study did not investigate overall survival.

**Table 7:** Breast cancer summary (*N* = 2)

Study name (year)	Study type/size	HALP threshold (method)	Outcome(s)	Summary
Importance of HALP Score in Breast Cancer and its Diagnostic Value in Predicting Axillary Lymph Node Status; Duran et al., (2022)	Retrospective *N* = 307	29.01 (ROC)	Predicting Axillary Lymph Node (ALN) Positivity in Breast Cancer Patients.	Although Low HALP <29.01 predicted increased rate of ALN positivity, HALP Score alone is not advised for ALN positivity.
Correlation of serum NLR, PLR and HALP with efficacy of neoadjuvant chemotherapy and prognosis of triple-negative breast cancer; Lou et al., (2022)	Retrospective *N* = 92	24.14 (ROC)	NAC efficacy and 3-year OS in triple-negative breast cancer.	Patients with High HALP >24.14 were more likely to have complete response to neoadjuvant chemotherapy and had improved 3-year survival.

Another study published by Lou et al., in 2022 investigated 92 triple-negative breast cancer patients to see if HALP had prognostic value in predicting response to neoadjuvant chemotherapy [62]. Patients who did achieve complete pathological response had a significantly higher HALP score (37.9 vs. 18.3, *p* < 0.001). Furthermore, an optimum cut-off of 24.14 was determined using ROC, which could predict complete pathological response to NAC with 81.1% sensitivity and 98.2% specificity. On multivariable binary logistic regression, Low HALP was shown to significantly predict poor NAC response (HR 0.518, 95% CI: 0.365–0.734, *p* < 0.001) when adjusting for TNM Stage III, lymph node metastasis, tumor size, and other hematological markers, platelet-to-lymphocyte ratio and neutrophil-to-lymphocyte ratio. Furthermore, patients with low HALP had significantly lower 3-year overall survival on Kaplan Meier Analysis (*p* < 0.001).

The range of HALP score cutoffs in studies on breast cancers was between 24.14–29.01 with a median of 26.575, which is lower than the median score cutoff of 31.2 in our review.

### Esophageal cancer (Table 8)

The first paper on esophageal cancer was published by Cong et al., in 2017, investigating a small sample of 39 inoperable squamous cell carcinoma male patients to see if HALP could function as a parameter to predict platinum-based definitive chemotherapy response and prognosis [64]. A cutoff of 48.34 was determined as the median HALP score of the population. Patients with low HALP had a complete chemotherapy response rate of 5% vs. 31.6% in the High HALP group (*p* = 0.044). Overall response rates, defined as complete response + partial response, to chemotherapy were 35% and 79% for low and high HALP, respectively (*p* = 0.010). Furthermore, High HALP patients were found to have significantly improved progression-free survival (24.7 months vs. 10.7 months, *p* = 0.041). However, there were no differences in overall survival.

**Table 8:** Esophageal and pharyngeal cancer summary (*N* = 5)

Study name (year)	Study type/size	HALP threshold (method)	Outcome(s)	Summary
The value of the combination of hemoglobin, albumin, lymphocyte and platelet in predicting platinum- based chemoradiotherapy response in male patients with esophageal squamous cell carcinoma; Cong et al., (2017)	Retrospective *N* = 39	48.34 (Median)	OS, Chemotherapy Response, and PFS in inoperable esophageal squamous cell carcinoma patients.	Patients with HALP >48.34 were shown to have increased complete response to chemotherapy and have improved PFS. No differences in OS were observed between High and Low HALP.
The preoperative hemoglobin, albumin, lymphocyte and platelet (HALP) score is a useful predictor in patients with resectable esophageal squamous cell carcinoma; Feng et al., (2021)	Retrospective *N* = 355	31.8 (ROC)	CSS in resectable patients undergoing curative resection for esophageal squamous cell carcinoma.	Patients with HALP >31.8 had superior 5-year CSS. Low HALP predicted worse OS when stratifying by Tumor Staging: TNM I, TNM II, and TNM III.
Preoperative maximal voluntary ventilation, hemoglobin, albumin, lymphocytes and platelets predict postoperative survival in esophageal squamous cell carcinoma; Hu et al., (2021)	Prospective *N* = 756	38.8 (ROC)	OS in patients who underwent radical esophagectomy with R0 resection.	Patients with HALP <38.8 had worse OS than patients HALP ≥38.8. Low HALP was associated with worse tumor characteristics.
Nutritional status at diagnosis is prognostic for pharyngeal cancer patients: a retrospective study; Wu et al., (2022)	Retrospective *N* = 319	44 (Mean)	OS and CSS based on HALP score at time of diagnosis in pharyngeal cancer patients.	Low HALP <44 predicted worse OS. HALP did not predict CSS, but was nearly significant.
Comprehensive risk evaluation for metachronous carcinogenesis after endoscopic submucosal dissection of superficial pharyngeal squamous cell carcinoma; Ogasawara et al., (2022)	Retrospective *N* = 144	40 (Median)	Predict the risk of metachronous carcinogenesis after endoscopic submucosal dissection in superficial pharyngeal squamous cell carcinoma.	Patients with Low HALP <40 were found to have over 3x increased risk of developing metachronous pharyngeal squamous cell carcinoma.

More recently, in 2021, Feng et al., showed HALP to be a useful predictor of cancer specific survival (CSS) in patients with resectable esophageal squamous cell cancer [79]. In this study of 355 patients, ROC found that an optimum threshold of 31.8 predicted 5-year CSS. For high HALP >31.8, CSS was 47.5% compared to 15.1% for low HALP ≤ 15.1% (*p* < 0.001). Furthermore, HALP was a significant predictor for 5-year CSS even when stratifying data by TNM I, TNM II, and TNM III, indicating it was able to outperform traditional TNM staging as a prognostic indicator.

A third paper by Hu et al., has investigated HALP in esophageal cancer [47]. This 2021 study investigated 756 patients who underwent radical esophagectomy with R0 resection. This study utilized the ROC curve to find an optimum HALP threshold of 38.8. Notably, HALP score was associated with tumor depth of invasion (*p* = 0.001) and tumor length (*p* < 0.001). Furthermore, patients with low HALP were shown to have worse overall survival on both univariable Cox regression analysis and multivariable Cox regression analysis adjusting for various clinical features such as tumor length, tumor differentiation, tumor invasion depth, Lymph node metastasis, and preoperative mechanical ventilation volume.

In these esophageal cancer studies, the range of HALP score cutoffs was between 31.8–48.34 with a median of 38.8, which is higher than the median score cutoff of 31.2 in our review.

### Pharyngeal cancer (Table 8)

There have been 2 studies to date investigating HALP’s prognostic ability in the setting of pharyngeal cancer, both being published in 2022. The first paper to do so was published by Wu et al., and considered 319 mostly male pharyngeal cancer patients, including nasopharyngeal, oropharyngeal, and hypopharyngeal carcinoma [67]. They chose a cut off of 44 based on the mean HALP of 44.2 in the sample. They found that patients with Low HALP had statistically significantly worse overall survival in univariate Cox regression analysis (*p* = 0.004). Furthermore, HALP was also an independent predictor after adjusting for gender, age, cancer site, cancer stage, and BMI.

The second study by Ogasawara et al., considered 144 patients with superficial pharyngeal squamous cell carcinoma (PSCC) to see if HALP could predict the risk of metachronous carcinogenesis after endoscopic submucosal dissection (ESD) [49]. In this study, metachronous carcinogenesis was defined as a second primary cancer occurring more than 1 year after the first ESD. HALP was dichotomized using an approximation of the median (median HALP = 37.9), in which a HALP of less than 40 was defined as low HALP. On univariable analysis, HALP score was found to be a significant predictor of metachronous PSCC, in low HALP patients had over 3x increased risk of developing metachronous PSCC.

In these pharyngeal cancer studies, the range of HALP score cutoffs was between 40–44 with a median of 42, which is higher than the median score cutoff of 31.2 in our review.

### Other tumors (Table 9)

In a 2021 study by Vlatka et al., the authors retrospectively queried 153 newly diagnosed diffuse large B-cell lymphoma patients receiving R-CHOP (Rituximab, Cyclophosphamide, Doxorubicin, Vincristine, and Prednisone) chemotherapy [80]. This study found that lower HALP was shown to be associated with unfavorable clinicopathological characteristics and a predictor of long term survival. This study used ROC to find 20.8 to be an optimal HALP cutoff. Patients with Low HALP ≤20.8 were found to have significantly more advanced stages of tumor (AA Stage III and IV) (*p* = 0.001), worse ECOG performance status (*p* = 0.001), higher CRP (*p* < 0.001), and increased LDH (*p* < 0.001). Low HALP patients were also more likely to experience B symptoms (*p* = 0.017), have bone marrow infiltration (*p* < 0.001), and worse response to treatment (*p* < 0.001). On multivariable analysis, low HALP patients were 3.7× more likely to fail treatment than high HALP patients when adjusting for age, gender, LDH, ECOG, Clinical Stage, the presence of B symptoms, and the International Prognostic Index (IPI). Patients with Low HALP also had significantly less 5-year survival (47.3% vs. 79.5%, *p* < 0.001). In fact, on multivariable Cox regression, patients were over 2.5× more likely to experience death during the 5-year time period if their HALP was low (*p* = 0.003), adjusting for age, gender, and IPI.

**Table 9:** Other tumors summary (*N* = 2)

Study name (year)	Study type/size	HALP threshold (method)	Outcome(s)	Summary
The hemoglobin, albumin, lymphocyte, and platelet (HALP) score is a novel prognostic factor for patients with diffuse large B-cell lymphoma; Vlatka et al., (2022)	Retrospective *N* = 153	20.8 (ROC)	5-year OS and EFS in newly diagnosed diffuse large B-cell lymphoma patients treated with R-CHOP.	Patients with Low HALP <20.8 has worse 5-year survival. Low HALP was also associated with several worse prognostic clinical and pathological factors.
The clinical significance of perioperative inflammatory index as a prognostic factor for patients with retroperitoneal soft tissue sarcoma; Matsui et al., (2022)	Retrospective *N* = 113	3.0 (Median)	OS in patients with retroperitoneal soft tissue sarcomas undergoing tumor resection.	Low HALP <3 was not associated with worse overall survival, although HALP was associated with worse overall survival in a subset of De-differentiated liposarcoma patients.

The first paper on HALP in the context of retroperitoneal soft tissue sarcoma was published in 2022 by Matsui et al., although HALP did not show any prognostic ability for overall survival [81]. This study investigated HALP amongst other scores including neutrophil-lymphocyte ratio (NLR), CRP-lymphocyte ratio (CLR), platelet-lymphocyte ratio (PLR), neutrophil-albumin ratio (NAR), CRP-albumin ratio (CAR), platelet-albumin ratio (PAR), prognostic nutrition index (PNI), and modified Glasgow Prognostic Score (mGPS). Interestingly, this study utilized a HALP score cutoff of 3.0, which was based on the median score of the study participants. It is important to note however, that this study presents a uniquely low HALP score compared to previous reports. However, the authors showed that HALP was significantly associated with worse overall survival in a subset of dedifferentiated liposarcoma (*p* = 0.0391).

Among these two studies evaluating other cancers, the range of HALP score cutoffs was between 3.0–20.8 with a median of 11.9, which is lower than the median score cutoff of 31.2 in our review.

### Non-cancer HALP applications

Although beyond the scope of the manuscript, it is worth noting that HALP has also been used to predict outcomes in acute ischemic stroke, antineutrophil cytoplasmic antibody-associated vasculitis, acute heart failure, and success of sleeve gastrectomy [82–85].

### Benefits for overall survival prognosis and other outcomes

Based on the details of the aforementioned literature, HALP appears to be a valuable prognostic indicator mainly of overall survival across various cancer subtypes. In most studies considered, overall survival was the primary outcome with remarkable prognostic ability. However, HALP has also been shown to predict other outcomes including progression-free survival, recurrence-free survival, and cancer-specific survival among others. Furthermore, HALP has even been shown to have potential in differentiating benign vs. malignant processes. It makes sense that HALP may best predict overall survival because HALP is considered to be a prognostic indicator investigating both the nutritional status and immune status, which are most closely related to the functional status of a patient, rather than a measure of tumor burden and presence of micrometastases in the body. However, HALP has still been used to predict the presence of lymph node metastasis (LNM) in several cancers in studies presented in this review, although with more limited efficacy. Furthermore, HALP has often been associated with other clinicopathological variables such as tumor size, depth of invasion, and other factors relevant to TNM staging. In several cases, HALP outperformed traditional TNM in predicting prognosis. HALP shows promise as a cost-effective and readily available adjunctive tool for the clinician to add to an ever-expanding toolbox of diagnostic and prognostic markers.

### HALP and tumor staging, grading, and size

Several studies have investigated HALP in relation to traditional TNM Staging and pathological grading. Chen et al., constructed a nomogram to predict gastric cancer outcomes incorporating HALP into TNM Staging, finding that overall prognostic ability was improved relative to TNM staging alone, as well as that HALP correlated with tumor size [2]. Furthermore, in training set, low HALP was able to delineate further worse prognosis in patients in a TNM III subset, although not in the validation set. Sun et al., also demonstrated that adding HALP to a TNM-based nomogram significantly improved the discriminative power on the TNM stagging system in biliary cancer [69]. Xu et al., showed that low HALP was significantly associated with poor tumor differentiation and higher TNM staging in pancreatic cancer [60]. Vlatka et al., has demonstrated that diffuse large B-cell patients with higher HALP were more likely be Stage I/II while lower HALP scores predicted increased rate of TNM Stage III/IV [80]. Duran et al., found low HALP breast cancer patients were also more likely to have more advanced clinical stage at time of surgery [61]. In cervical cancer, Leetanaporn et al., found lower HALP predicted higher tumor stage and larger tumor size [48]. Njoku et al., found low HALP was associated with worse FIGO stage and worse differentiation in endometrial cancer [63]. Peng et al., showed that creating a nomogram with HALP better predicted radical cystectomy and radical nephrectomy outcomes than traditional TNM Staging [42, 43]. In prostate cancer, HALP has also been shown to correlate with Gleeson grade, and the combination of HALP with Gleeson grade was an independent risk factor with prostate-specific antigen progression free survival [66]. In non-metastatic upper tract urothelial carcinoma undergoing radical nephroureterectomy, Gao et al., showed that HALP score correlated with tumor stage, grade, and size. The HALP score could also stratify patients for survival under different pathologic T stages in the subgroup analysis [45]. Zhai et al., and Wei et al., both showed that HALP was correlated to tumor size in non-small cell lung cancer.

Interestingly, in esophageal squamous cell carcinoma, both Cong et al., and Hu et al., demonstrated HALP was not associated with TNM staging. At the same time, Hu also found HALP was not associated with tumor differentiation [47, 64]. However, Hu et al., found lower HALP was associated with deeper depth of invasion and larger tumor size. Feng et al., did suggest that in in esophageal squamous cell carcinoma, low HALP predicted worse TNM staging, but not less tumor differentiation [79]. Low HALP predicted worse outcomes within TNM I, II, and III, further suggesting that HALP can differentiate prognosis in patients with the same TNM stage. Low HALP also predicted larger tumor size.

### HALP and chemotherapy/radiation

Several studies have evaluated the relationship between HALP scores and chemotherapy/radiation. Understanding this relationship further could help future clinicians determine optimal treatments for patients using HALP as a prognostic biomarker. In Cong et al., low HALP scores (<48.34) in esophageal squamous cell carcinoma (ESCC) male patients receiving docetaxel with cisplatin or carboplatin were associated with lower chemoradiotherapy response rates (*p* = 0.01). This suggests that measuring HALP before treatment could help predict platinum-based chemoradiotherapy response of tumors and progression free survival [64]. Another study by Vlatka et al., evaluating HALP and treatment in patients with diffuse large B-cell lymphoma found that a low HALP score was associated with a worse response to R-CHOP-21 (rituximab, cyclophosphamide, doxorubicin, vincristine, and prednisone) and R-CHOP like treatment regimens (*P* < 0.001). This study showed that low HALP patients were 3.7× more likely to fail this treatment than high HALP score patients when adjusting for factors such as age, gender, LDH, ECOG, Clinical Stage, the presence of B symptoms, and the International Prognostic Index (IPI). The higher risk of treatment failure with a low HALP score indicates the value this pre-treatment biomarker can have on patient clinical outcomes. Higher HALP scores had better responses in complete and partial remission to treatment [80].

Furthermore, one study by Shen et al., showed that small-cell lung cancer patients over the age of 65 with high HALP scores (>25.8) had increased PFS following etoposide-based first-line treatment combined with platinum [50]. In patients with triple-negative breast cancer (TNBC), a low HALP score suggested poorer prognosis in those who did not benefit from neoadjuvant chemotherapy (NAC) and was a risk factor for reduced NAC efficacy overall [62]. Interestingly, Leetanaporn et al., found that a low HALP score (<22.2) in patients with locally advanced cervical cancer had a higher probability of receiving radiation alone than concurrent chemoradiation (*p* < 0.001). The ability to stratify patients this way may prove helpful as a clinical prognostic factor before and after radiation and concurrent chemoradiation treatments [48]. Moreover, HALP was assessed to predict pembrolizumab’s efficacy in 54 patients with advanced metastatic urothelial cancer previously treated with systemic chemotherapy. Although a low HALP score was associated with poorer initial tumor response to pembrolizumab, this finding was not statistically significant (*p* < 0.064) [77]. These studies emphasize the need for further investigation into the utilization of HALP for predicting treatment response and show promise in how clinicians may integrate HALP into practice.

### Threshold determination and other limitations

One limitation of HALP is that each study utilized one of three different methods to find the “optimal threshold” of HALP. The three main methods of determining HALP thresholds were using: X-tile software by the Yale Rimm Lab, ROC curves, and median/mean approximation. It is worth noting that X-tile and ROC curves are designed to yield statistically significant results. For example, X-tile software seeks to dichotomize a variable to produce the most significant Kaplan-Meier curve possible [86] and as such it is expected that HALP will be significant. Similarly, ROC curves seek to maximize specificity and sensitivity of a variable to determine the optimal threshold [87]. As such, this should be considered when interpreting the optimal thresholds presented for HALP. The result of the heterogeneity in identifying optimal thresholds is that thresholds are often different from one cancer to another and within cancer subtypes. Furthermore, thresholds may differ based on outcome (i.e. OS vs. RFS vs. LNM). This inherently may limit the general use of HALP, as there are no defined thresholds in the healthy population, but rather many different optimizations based on very specific cohorts of cancer patients and outcomes.

Furthermore, HALP may be limited in patients with certain comorbidities that directly impact the individual elements of the HALP score – Hemoglobin, albumin, lymphocytes, and platelets. While HALP is a prognostic indicator and often correlates with comorbidities, it is unclear if HALP can be used equally between patients with different comorbidities. For example, patients with liver disease have a reduction in albumin synthesis due to a reduction in hepatocyte mass, resulting low serum albumin scores [88]. Patients with a primary liver tumor or metastasis to the liver would be expected to lose greater hepatocyte function than in other tumor types. Moreover, patients who other forms of liver disease, such as cirrhosis, non-alcoholic fatty liver disease, alpha-1 antitrypsin deficiency, hemochromatosis, Wilson’s disease, among others can all impact hepatic function, and thus the HALP score [89–91]. It has not been studied to determine if HALP can be applied effectively in these patient subtypes.

This limitation also applies to other comorbidities. For example, patients with sickle cell disease of thalassemia may see reductions in their hemoglobin score [92, 93]. Primary polycythemia vera patients will have an increased hemoglobin. Certain tumors such as renal cellular carcinoma release erythropoietin, while hepatocellular carcinoma is known to cause erythrocytosis [94, 95]. In addition, lymphomas and leukemias may distort the lymphocyte count relative to other cancer types [96, 97]. Patients with myelofibrosis and other diseases of the bone marrow may see reductions in hemoglobin, lymphocytes, and platelets [98]. Patients actively on chemotherapy may also experience pancytopenia. Ultimately, any clinician utilizing the HALP score must consider these comorbidities when interpreting the HALP score and utilize clinical judgment when applying HALP to an individual patient.

## FUTURE APPLICATIONS OF HALP

While HALP has shown promise as a prognostic indicator, it remains unclear how HALP may be incorporated into clinical practice. For example: should patients with low HALP be treated with immunonutritional therapy during chemotherapy or pre/peri-operatively? Nutritional and Immuno-supportive therapy has been a debated topic for some time. Ziętarska et al., found that nutritional support did not necessarily result in better tolerated chemotherapy, although improvements in albumin and prealbumin were observed [99]. However, this study did not consider long-term overall survival. Jie et al., did find that abdominal surgical patients who received a minimum of 7 days of parenteral nutrition or enteral nutrition before surgery benefited from a shorter postoperative hospital stay than the control group (13.7 ± 7.9 versus 17.9 ± 11.3 d, *P* = 0.018) [100]. As such, there may be a role for preoperative nutritional support in oncology patients who require surgery and are determined at-risk of malnutrition. Furthermore, preoperative oral arginine and n-3 fatty acid supplementation has been shown to improve the immunometabolic host response and outcome after colorectal resection for cancer, showing decreased infection rate, and improved gut oxygenation and microperfusion [101]. L-arginine supplementation and other immunonutritional support has also been shown to decrease infection rates and lower inflammatory response in radical cystectomy patients [102, 103]. As of now, there is no defined standard of care for immune-nutritional supplementation during cancer treatment. By virtue of being a strong prognostic indicator, HALP shows potential in guiding the way in informing when clinicians should use immune-nutritional supplementation. It remains to be seen if immune-nutritional supplementation would increase the HALP score and if any potential change would ultimately impact outcomes. Future studies should focus on optimizing the delivery of immune-nutritional therapy based on HALP as well as if doing so improved outcomes. Lastly, HALP calculation timing should be standardized in patients scheduled for surgery and/or systemic treatment. Currently, there is no standard for when HALP should be collected whether immediately post-diagnosis, pre-chemotherapy, or pre-surgery, among other possibilities, which have all been used as HALP collection times.

## CONCLUSIONS

HALP has shown an ability to be a useful prognostic biomarker in various cancers, including gastrointestinal, lung, urinary tract, gynecological, otorhinolaryngological, among others. The median HALP score cutoff across all tumor subtypes in this review is 31.2, with some cancer subtypes having higher or lower median scores, highlighting the heterogeneity of the HALP score. To date, HALP has only shown theoretic prognostic ability, and has not yet been used in clinical practice to tailor treatment for those at risk for immunonutritional deficiencies.

## Abbreviations

ACD: Anemia of Chronic Disease
AJCC: American Joint Committee on Cancer
ALN: Axial Lymph Node
ASA: American Society of Anesthesiology
AUC: Area Under the Curve
BPH: Benign Prostate Hyperplasia
CA199: Carbohydrate Antigen 19-9
CAR: CRP-Albumin Ratio
CCRT: Concurrent Chemoradiation Therapy
CEA: Carcinoembryonic Antigen
CLR: CRP-Lymphocyte Ratio
CRP: C-Reactive Protein
CSS: Cancer-Specific Survival
DFS: Disease-Free Survival
ECOG: Eastern Cooperative Oncology Group score
EFS: Event-Free Survival
ESCC: Esophageal Squamous Cell Carcinoma
ESD: Endoscopic Submucosal Dissection
FIGO: International Federation of Gynecology and Obstetrics
HALP: Hemoglobin, Albumin, Lymphocyte, Platelet score
IPI: International Prognostic Index
LACRC: Locally Advanced Colorectal Cancer
LDH: Lactate Dehydrogenase
LNM: Lymph Node Metastasis
LVSI: Lymphovascular Space Invasion
mGPS: Modified Glasgow Prognostic score
NAC: Neoadjuvant Chemotherapy
NAR: Neutrophil-Albumin Ratio
NLR: Neutrophil-to-lymphocyte Ratio
NSCLC: Non-Small Cell Lung Cancer
OS: Overall Survival
PAR: Platelet-Albumin Ratio
pCA: Prostate Cancer
PECAM-1: Platelet-Endothelial Adhesion Molecule
PFS: Progression-Free Survival
PLR: Platelet-to-Lymphocyte Ratio
PNI: Prognostic Nutrition Index
PSCC: Pharyngeal Squamous Cell Carcinoma
PSGL-1: P-selectin Glycoprotein Ligand
RCC: Renal Cell Carcinoma
R-CHOP: Rituximab, Cyclophosphamide, Doxorubicin, Vincristine, and Prednisone
RFS: Recurrence-Free Survival
ROC: Receiver Operating Characteristics
RT: Radiation Therapy
SCLC: Small-Cell Lung Cancer
TCIPA: Tumor-Cell Induced Platelet Aggregation
TNBC: Triple-Negative Breast Cancer
VEGF: Vascular Endothelial Growth Factor
