# Addendum: What is hemoglobin, albumin, lymphocyte, platelet (HALP) score? A comprehensive literature review of HALP’s prognostic ability in different cancer types

**DOI:** 10.18632/oncotarget.28485

**Published:** 2023-08-07

**Authors:** Christian Mark Farag, Ryan Antar, Sinan Akosman, Matthew Ng, Michael J. Whalen

**Affiliations:** ^1^Department of Urology, George Washington University School of Medicine, Washington, DC 20052, USA; ^2^Department of Surgery, George Washington University School of Medicine, Washington, DC 20052, USA

**This article has an addendum:** No external funding was obtained for this article.

Original article: Oncotarget. 2023; 14:153–172. 153-172. https://doi.org/10.18632/oncotarget.28367

